# The DREAMS core package of interventions: A comprehensive approach to preventing HIV among adolescent girls and young women

**DOI:** 10.1371/journal.pone.0208167

**Published:** 2018-12-07

**Authors:** Janet Saul, Gretchen Bachman, Shannon Allen, Nora F. Toiv, Caroline Cooney, Ta’Adhmeeka Beamon

**Affiliations:** Office of the U.S. Global AIDS Coordinator and Health Diplomacy, U.S. Department of State, Washington, DC, United States of America; Desmond Tutu HIV Centre, SOUTH AFRICA

## Abstract

In sub-Saharan Africa, adolescent girls and young women (AGYW) are 5 to 14 times more likely to be infected with HIV than their male peers. Every day, more than 750 AGYW are infected with HIV. Many factors make girls and young women particularly vulnerable to HIV, including gender-based violence, exclusion from economic opportunities, and a lack of access to secondary school. The President’s Emergency Plan for AIDS Relief (PEPFAR) is dedicating significant resources through the Determined, Resilient, Empowered, AIDS-free, Mentored, and Safe (DREAMS) partnership to impact the lives of women and girls based on PEPFAR’s mission to help countries achieve epidemic control of HIV/AIDS. The data show that new HIV infections must be reduced in AGYW, or the global community risks losing the extensive progress made towards reaching epidemic control. With support from PEPFAR and private sector partners—the Bill & Melinda Gates Foundation, Gilead Sciences, Girl Effect, Johnson & Johnson and ViiV Healthcare, DREAMS works together with partner governments to deliver a core package of interventions that combines evidence-based approaches that go beyond the health sector, addressing the structural drivers that directly and indirectly increase girls’ HIV risk. Not only is DREAMS an effort to reduce new HIV infections, but it aims to reduce other critical vulnerabilities such as gender-based violence. When girls and young women thrive, the effects are felt throughout their families, communities and countries.

## Introduction

Despite substantial declines in the number of new HIV infections, the epidemic among females aged 15–24 in sub-Saharan African countries remains uncontrolled, with 2/3 of new infections occurring in this population, or an estimated 280,000 new infections annually [1, 2]. HIV prevalence for female youth aged 15–24 is consistently higher than their male counterparts [3, 4] with adolescent girls and young women (AGYW) up to 14 times more likely to become HIV-infected than their male counterparts in Malawi, Zambia and Zimbabwe [5–7].

Simultaneously, the population of young females in sub-Saharan Africa (SSA) is expected to double from 100 million (in 1990) to 200 million by 2020 [8], suggesting the potential for millions of new infections if current epidemiological trends are not reversed. The alarming combination of this doubling population, coupled with elevated infection rates, demonstrates an urgent need for programs designed to avert new infections in AGYW in areas with high HIV burden.

A myriad of factors are associated with vulnerability to HIV among AGYW. Recent systematic reviews cite a history of sexually transmitted infections (STIs), alcohol use, multiple sex partners, early marriage, being out of school, inconsistent condom use, and engaging in transactional sex as factors that are associated with HIV acquisition for young women [9, 10]. Moreover, experiences with violence are inextricably linked to HIV risk among AGYW [3, 11, 12]. According to the PEPFAR-supported Violence Against Children Surveys (VACS) in Eswatini, Kenya, Tanzania, and Zimbabwe, one in three females report experiencing some form of sexual violence during childhood [13].

To control the epidemic among this highly vulnerable group, the United States President’s Emergency Plan for AIDS Relief (PEPFAR) announced the Determined, Resilient, Empowered, AIDS-free, Mentored and Safe (DREAMS) Public/Private Partnership in 2014. DREAMS began in ten countries, representing over half of all infections occurring among AGYW globally. These included Eswatini, Kenya, Lesotho, Malawi, Mozambique, South Africa, Tanzania, Uganda, Zambia, and Zimbabwe. In partnership with private sector partners—the Bill & Melinda Gates Foundation, Girl Effect, Johnson & Johnson, Gilead Sciences, and ViiV Healthcare—PEPFAR’s DREAMS partnership delivers a core package of interventions combining evidence-based approaches that go beyond the health sector to directly address the structural drivers that increase HIV risk.

Although individual interventions have shown promise, no single intervention has emerged that can avert the majority of new HIV infections in AGYW [14–16], possibly because of the complex constellation of factors that place them at risk. This underscores the need to develop comprehensive packages of social, economic and biomedical interventions to both reduce girls’ vulnerability to HIV and increase their agency [17–19]. This article describes PEPFAR’s approach to preventing new HIV infections among females 15–24 through DREAMS and reviews the rationale for this approach. Because the objective of DREAMS is to reduce HIV risk and lower incidence rates in 15–24 year-old girls and women, it is critical to also intervene with younger girls (ages 10–14) with prevention interventions before they reach 15 years old, when risk increases substantially.

## The PEPFAR strategy for adolescent girls and young women

**Fig 1:**
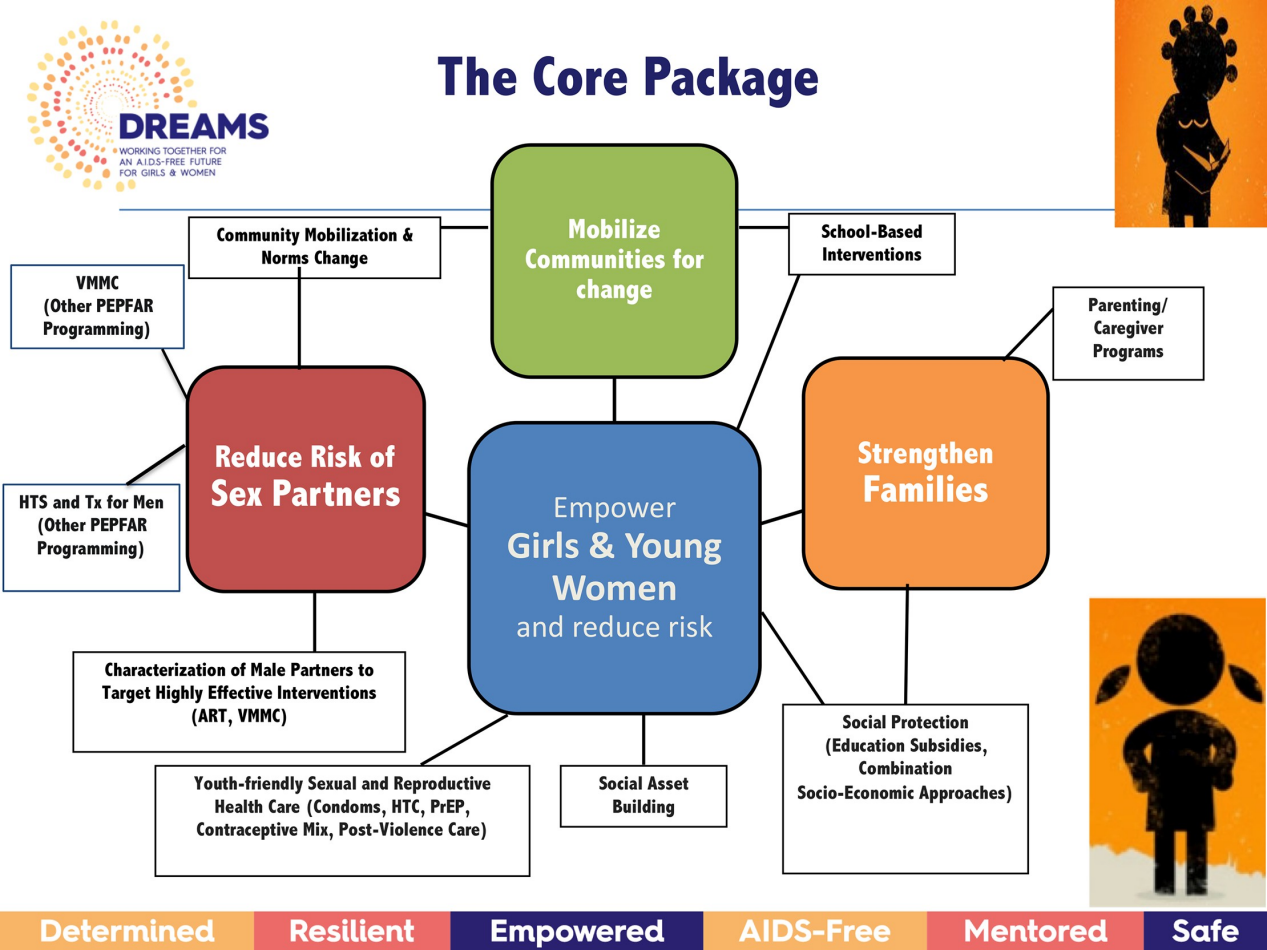
The core package.

To respond to the realities in which AGYW are living, the DREAMS Partnership requires a multi-faceted, integrated response from the health, education, psychosocial, economic and civil society/community sectors. To limit the interventions implemented by DREAMS, several selection criteria were utilized to identify and classify those most likely to impact HIV risk behaviors and incidence among AGYW. Categories of interventions (e.g., parenting/caregiver programs, combination socioeconomic approaches), were rated with regard to quality of evidence (e.g., number and rigor of evaluations) and consistency of results across studies using a documented method for grading the quality of available scientific evidence [20]. Prudent practices, defined as interventions, policies, or delivery methods that have not been extensively evaluated but support the proposed evidence-based strategies, were also considered (e.g., making services adolescent friendly). The resulting DREAMS components, or core package of interventions and the associated sub-interventions, are represented in *Fig 1*. The core package includes interventions that: (1) empower adolescent girls and young women and reduce their risk, (2) strengthen the families of AGYW, (3) mobilize communities for change and (4) reduce the risk of men who are likely to be male sex partners of AGYW. Evidence-based violence prevention interventions, most of which were created in or adapted for low and middle income countries, are integrated into all components of the package.

**Fig 2:**
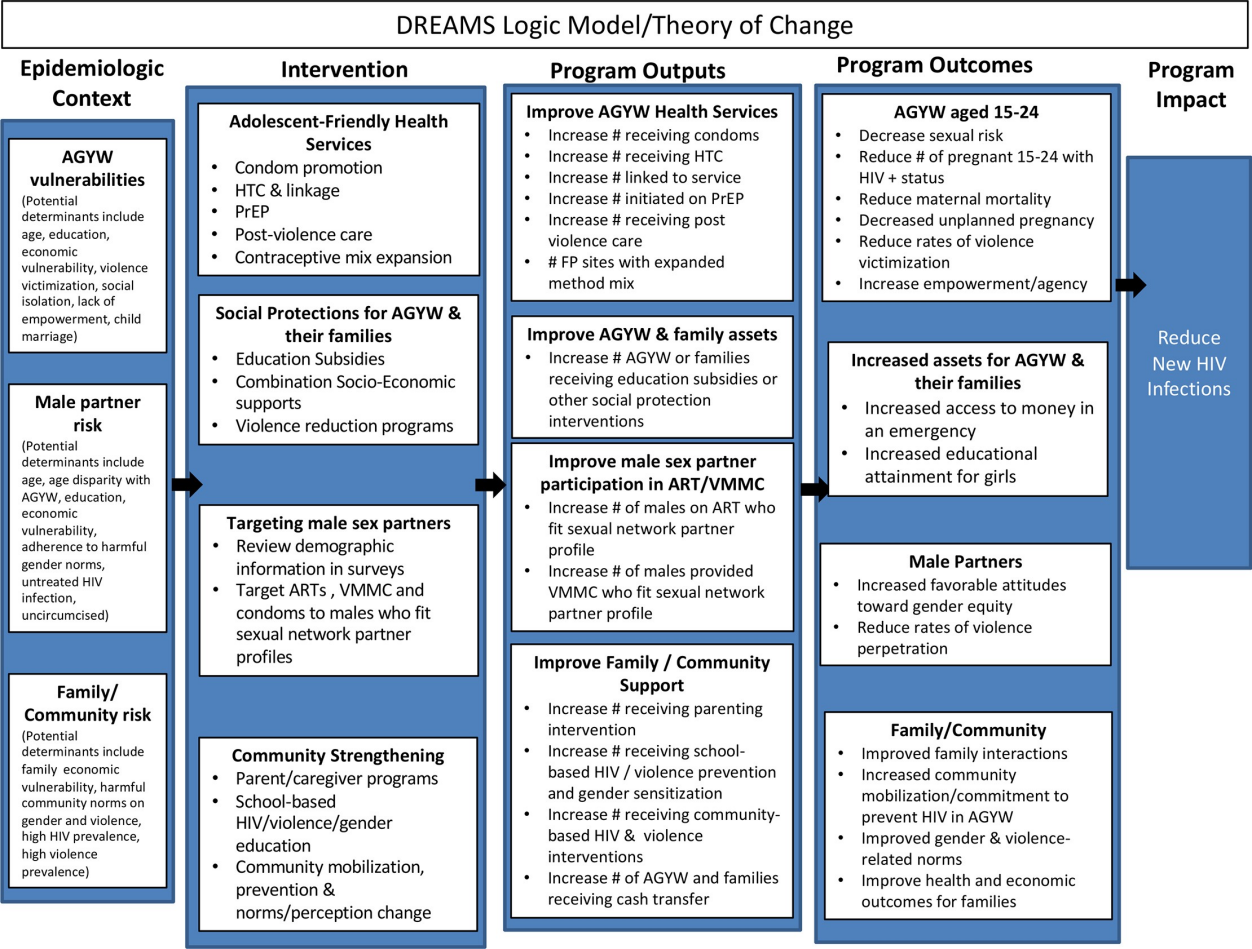
DREAMS logic model/theory of change.

The DREAMS logic model is presented in Fig 2. This logic model represents the DREAMS theory of change, and is linked to the DREAMS core package of interventions and DREAMS monitoring and evaluation framework. The ultimate goal of DREAMS is to reduce new HIV infections among AGYW 15–24 years of age. The theory of change posits that when multiple interventions from the core package are delivered to vulnerable AGYW (i.e., layering of interventions), those interventions will act synergistically to change both the program outcomes and impact. The general pathways through which this is expected to be achieved is shown in the logic model by the program outputs and outcomes. Some of the changes to HIV acquisition are likely to be direct and proximal (e.g., through provision of PrEP), whereas some are likely to be indirect and more distal (e.g., decreasing AGYW’s rates of violent victimization).

The following section describes each component of the DREAMS core package of interventions. Illustrative examples of specific evidence-based programs or curricula are given, based on those that are listed in the DREAMS guidance.

1. **Empower Adolescent Girls and Young Women (AGYW) and Reduce their Risk:** These interventions aim to empower girls and reduce risk for HIV, unintended pregnancy, and violence.

Condom promotion and provision: Condoms are highly effective at preventing STIs, including HIV, when used correctly and consistently [21–24]. The promotion and provision of male and female condoms is offered throughout the DREAMS core package to AGYW, male sex partners, and community members to increase consistent use and availability. DREAMS facilitates a youth-friendly environment and provides interpersonal counseling to ensure that AGYW understand the importance of consistent condom use in protecting their sexual and reproductive health and in dual method use for protection from both pregnancy and STIs.

Pre-exposure prophylaxis (PrEP): Oral PrEP refers to the daily ingestion of antiretroviral drugs formulated into a single pill to prevent HIV acquisition in negative persons. It is an autonomous, highly effective intervention if adherence is maintained [25–31] and represents a new opportunity for reducing HIV incidence in AGYW at elevated risk of infection [32–34]. PrEP is provided in the context of the full DREAMS core package of services and in settings where there is support and buy-in from partner governments.

Post-violence care, including post-exposure prophylaxis (PEP): Studies show a significant association between violence and HIV status in women, with women who experience violence being at 3-fold increased risk for HIV and other STIs [3, 11]. Country teams implement a minimum set of services as indicated in WHO normative guidance [35]. As part of that set of services, the provision of post-exposure prophylaxis (PEP) is strongly supported as a highly efficacious HIV prevention method after sexual assault and should be administered within 72hrs [36–40].

HIV testing services (HTS): This essential intervention seeks to increase knowledge of serostatus among AGYW and their partners and to enhance HIV knowledge. Early diagnosis facilitates earlier linkage to care and initiation on ART [41], and data suggest that HTS may have prevention benefits among youth [42]. The importance of linking to appropriate services (i.e. PrEP, PEP, etc.) from the testing platform cannot be underemphasized. DREAMS facilitates strategies, such as mobile vans, self-testing, and testing after-hours and on holidays, to ensure that AGYW and their partners are reached with HTS through facility and community-based platforms and are then appropriately linked to HIV prevention or treatment services.

Expand & improve access to voluntary, comprehensive family planning (FP) services: AGYW in low-income countries experience high rates of early pregnancy which is associated with lower educational attainment and socioeconomic status [43–45], making AGYW more vulnerable to transactional sex, gender-based violence, and potentially HIV [46–49]. Sexual violence can lead to unplanned pregnancy. Although PEPFAR does not purchase FP commodities, DREAMS provides counseling and education about the mix of available contraceptive methods as a means to prevent both HIV and pregnancy, with an emphasis on dual method use [50–53].

Social asset building: The most vulnerable AGYW often lack strong social networks, such as relationships with peers and adults who can offer emotional support, information, and material assistance. Although social asset building has not been linked directly to decreases in HIV acquisition, interventions that build social capital have been shown to increase agency and empowerment among AGYW [54–56]. In order to assist AGYW in making important connections and in receiving support from each other and near-peer role models, DREAMS promotes the prudent practice of holding small, female mentor-led group meetings in safe, public spaces on a regular basis, often weekly. One model for DREAMS social asset building used by several county teams is Safe Spaces through which AGYW receive support, but also serve as a programmatic hub to link girls to additional DREAMS interventions and services [57–59]. Thus, the social asset building component of the core package is often a critical part of the DREAM layering process.

2. **Strengthen Families:** These interventions aim to economically strengthen the families of AGYW, and improve their ability to positively and effectively parent.

Parenting/caregiver programs: Having positive relationships with parents, caregivers, or other caring adults is a consistent protective factor for young women and girls against a variety of negative health and social outcomes [60]. DREAMS facilitates parent/caregiver programs that increase caregivers’ knowledge, skills, and comfort with talking to their children about sexual health and monitoring their children’s activities. These interventions have shown promise in changing risky sexual behavioral patterns among youth, including delayed sexual debut, condom use, and decreased exposure to negative outcomes such as violence and abuse. Most country teams are implementing either the Families Matter Program (FMP) or Sinovoyu [61, 62].

Educational subsidy for transition to and attendance of secondary school: Female students are especially vulnerable to school dropout and are more likely than boys to never attend school at all [63]. Educational subsidies are an effective intervention for keeping girls in school [32, 33] and are correlated with reduced risky sexual behaviors, higher HIV testing, reduced likelihood of early marriage [64], decreased school dropout rates and other negative outcomes among female adolescents [61, 65–71]. When conditioned on school attendance, cash transfers have been shown to keep girls in school primarily in geographic settings with high levels of economic barriers [15]. Additional research suggests a correlation between secondary schooling [72] and HIV negative status, and that additional secondary schooling may be nearly as cost-effective for HIV prevention as PrEP [71, 73, 74].

Combination socio-economic approaches: Stand-alone economic empowerment interventions demonstrate variable effectiveness [75–77]. Combining economic and social empowerment interventions have demonstrated more consistent effects on both behavioral and violence outcomes [78–85]. Economic strengthening interventions, especially low-cost savings-led approaches, within PEPFAR’s orphans and vulnerable children (OVC) programs are being leveraged for this initiative. The social empowerment literature-supported interventions include discussion groups on gender-based violence/intimate partner violence (GBV/IPV) and couples communication [81, 82], mentoring [79, 84], and comprehensive, evidence-based HIV prevention curricula [76, 78, 80, 83]. An example of what some country teams are implementing for this component is IMAGE, a program that combines microfinance with a training curriculum on HIV prevention, gender norms, and gender-based violence. IMAGE has been shown to decrease intimate partner violence among program participants [86].

3. **Mobilize Communities for Change:** These interventions aim to educate communities of AGYW, including boys and young men, and mobilize communities for change to keep girls HIV free and safe from violence.

School-based HIV and violence prevention: DREAMS programs deliver school-based HIV and violence prevention in order to provide accurate information, provide referrals to health centers for services not provided in school, and to build prevention skills among large numbers of young people in a community. HIV/AIDS and sexuality education that meets established standards has been shown to delay sexual initiation, increase use of condoms and/or oral contraceptives, and decrease number of sex partners, frequency of unprotected sex, STIs and pregnancy [20, 87–92]. Furthermore, sexuality education curricula that address gender and power relations are associated with improved behavioral outcomes, including significantly lower rates of STIs and unintended pregnancy. Effective school-based programs also exist to decrease both victimization and perpetration of violence. All curricula used in DREAMS should be developmentally appropriate with a stronger focus on primary prevention of sexual violence and HIV risk among 10–14 year olds, with an increasing focus on risk reduction (i.e., methods for preventing HIV, STIs, and pregnancy) for older age groups.

Community mobilization/norms change: Community mobilization provides an essential support framework for HIV prevention programs [93–95] and engages boys/men [96], community leaders and the broader community in addressing social norms that increase HIV risk for AGYW. Community mobilization efforts in related areas, like GBV prevention, have shown a significant impact on norms change, a decrease in violent victimization and perpetration [97, 98], and an increase in empowerment [62]. Community mobilization in DREAMS engages men and boys, especially opinion leaders, in community conversations about HIV, gender norms, sexuality, relationships, joint decision-making and alcohol use. DREAMS implements curricula with a participatory learning component that focuses on building skills and a community-level awareness and ownership of HIV risk reduction (e.g., SASA! and SHARE).

4. **Reduce the Risk of Sexual partners of AGYW:** These interventions aim to decrease the risk that male sexual partners pose to AGYW. While not described in this article, PEPFAR’s core activities include a focus on men in the same geographic locations as DREAMS including HIV testing, treatment and voluntary medical male circumcision (VMMC).

Characterization of male sexual partners of AGYW to better target HIV services: To prevent HIV among AGYW, their sex partners must have access to highly effective HIV services including HTS, VMMC [99–105], condoms, and treatment for viral suppression [106]. Therefore, it is imperative to understand who the “typical” sexual partners of AGYW are [107]. DREAMS encourages examination of available data through surveys such as the VACS, Demographic Health Surveys (DHS), and Population-based HIV Impact Assessments (PHIAs) to understand the characteristics of AGYW’s male sex partners to better target HIV services for men.

It should be noted that there are several interventions that are intentionally not included in the DREAMS core package. Some are not included due to either inconclusive research findings or findings of ineffectiveness (treatment for Schistosomiasis, abstinence-only education, credit- or income-based approaches to income strengthening and stand-alone youth centers). Other interventions are available through core PEPFAR activities (HIV care and treatment for pregnant girls, young mothers and their male sexual partners and VMMC for men).

### Principles of DREAMS implementation

#### Layering of interventions

DREAMS seeks to reduce HIV risk and increase agency of AGYW using a layered approach. The layering of interventions is a fundamental principle of DREAMS, based on research from related fields demonstrating that addressing multiple needs of young people will have greater impact on risk behaviors than any single intervention. At the individual level, layering is defined as providing multiple interventions from the DREAMS core package to each DREAMS recipient. While PEPFAR programs were overlapping some interventions in the past, especially through the OVC program, layering of this type and scale was not occurring for vulnerable AGYW. Layering in DREAMS has required multiple agencies, implementing partners, and technical areas to collaborate more intensely to ensure that AGYW get the right interventions layered to meet their diverse needs. The exact package of interventions that should be layered depends on three factors: 1) which interventions and services are included in the country’s DREAMS program (there is some variation based on context, laws, and policies—see Table 1); 2) age of the AGYW (10–14, 15–19, 20–24); and 3) needs of the AGYW (e.g., is the AGYW sexually active, has she experienced violence). In addition to the individual level, layering also takes into account contextual level interventions that are not delivered directly to an AGYW but from which she may benefit such as community mobilization/norms change programs and parent/caregiver programs. Layering also goes beyond simple referrals to ensuring actual linkage from one intervention to another.

**Table 1:** Implementation of DREAMS core package, by country.

Empower AGYW and Reduce Their Risk							Mobilize the Community		Strengthen Families			Decrease Risk in Sex Partners	
**Eswatini**	**X**	**X**	**X**	**X**	**X**	**X**	**X**	**X**	**X**	**X**	**X**	**X**	**X**
**Kenya**	**X**	**X**	**X**	**X**	**X**	**X**	**X**	**X**	**X**	**X**	**X**	**X**	**X**
**Lesotho**	**X**	**X**	**X**	**X**	**X**	**X**	**X**	**X**	**X**	** **	**X**	**X**	**X**
**Malawi**	**X**	**X**	**X**	**X**	**X**	**X**	**X**	**X**	**X**	**X**	**X**	**X**	**X**
**Mozambique**	**X**	**X**	**X**	**X**	**X**	**X**	**X**	**X**	**X**	**X**	**X**	**X**	**X**
**South Africa**	**X**	**X**	**X**	**X**	**X**	**X**	**X**	**X**	**X**	**X**	**X**	**X**	**X**
**Tanzania**	**X**	**X**	** **	**X**	**X**	**X**	**X**	**X**	**X**	**X**	**X**	**X**	**X**
**Uganda**	**X**	**X**	**X**	**X**	**X**	**X**	**X**	**X**	**X**	** **	**X**	**X**	**X**
**Zambia**	**X**	**X**	**X**	**X**	**X**	**X**	**X**	**X**	**X**	**X**	**X**	**X**	**X**
**Zimbabwe**	**X**	**X**	**X**	**X**	**X**	**X**	**X**	**X**	**X**	**X**	**X**	**X**	**X**
** **	**Condom Promotion & Provision**	**HIV Testing & Counseling**	**PrEP**	**Post Violence Care**	**Social Asset Building**	**Increase Contraceptive Method Mix**	**School Based HIV & Violence Prevention**	**Community Mobilization & Norms Change**	**Parenting/ Caregiver Programs**	**Education Subsidies**	**Combination Socio-Economic Approaches**	**Characterization of Male Sex Partners**	**Linking Male Partners to Services**

#### Quality of implementation

The DREAMS core package specifies what evidence-based programs and services should be implemented for each component of the package, but how these interventions are implemented is also critically important. Country teams are encouraged to implement each intervention based on normative guidance (e.g., for clinical interventions) or based on the delivery methods used when the intervention was originally developed and evaluated. This means that interventions delivered as part of DREAMS are a combination of peer-led, facilitator-led, health-care-worker-led, participatory, small groups and large groups. Another way that DREAM supports quality implementation is through the training of implementers. Trainings for some interventions were conducted with implementing partners from multiple countries (e.g., a SASA! training in Uganda), whereas other trainings are held in each country implementing a program (e.g., Families Matter Program). In addition to training on the content and delivery of specific programs, trainings are offered on how to successfully engage and approach AGYW. Examples include training on how to provide non-judgmental, adolescent-friendly clinical services. Training for teachers is also being supported through collaborations with Ministries of Education and Health to ensure that teachers are comfortable and confident delivering HIV prevention curricula.

#### Stakeholder engagement

Critical stakeholders for DREAMS include government, civil society including faith-based groups, other donors, and AGYW themselves. Given the nature of the DREAMS core package, multi-sectoral stakeholder buy-in, political will, and shared responsibility are essential for success and sustainability as this is likely dependent on integration into existing government-supported systems and structures. In some instances, important policy, structural, and system reforms within the current health, education, and judicial systems are necessary to ensure the sustainable impact of these interventions. For example, government policies/regulations ensuring universal access to secondary education, ending restrictions on contraception access, increasing access to comprehensive HIV prevention education in schools, prosecuting perpetrators of gender-based violence, and prohibiting child marriages can all be leveraged as part of a partnership with the government in reaching DREAMS goals. A significant shift has occurred in the number of countries implementing PrEP for AGYW. DREAMS country teams worked closely with partner governments as they shifted their policy landscape for PrEP. Other stakeholders who have been critical to moving oral PrEP for AGYW forward in DREAMS countries include DREAMS partner Gilead Sciences, local advocates, and young women themselves. Most importantly, engagement with DREAMS recipients, as well as AGYW living with HIV, has made DREAMS programming more responsive to the myriad needs of AGYW. A number of beneficiaries have been promoted to DREAMS Ambassadors—they recruit AGYW to participate in DREAMS, advise PEPFAR staff, and speak about DREAMS to government and civil society. Donors and country governments must engage with, ask of, and listen to AGYW about how best to keep them HIV free.

### Monitoring and evaluation of DREAMS

The DREAMS partnership uses several approaches to monitor program implementation and measure impact. Various monitoring and evaluation (M&E) activities aim to monitor how well DREAMS is being implemented and if the lives of AGYW in DREAMS districts are improved. The sub-questions that are being explored regarding implementation include: 1) Is DREAMS identifying and reaching the right AGYW?; 2) Are implementing partners reaching their targets?; and 3) Are DREAMS interventions being successfully layered? The sub-questions on impact include: 1) Is there a reduction in new diagnoses among females 15–24 in DREAMS districts?; and 2) Are there changes in other important outcomes (e.g., secondary school enrollment and completion, violence, early sexual debut, early pregnancy). These questions align with the DREAMS theory of change as expressed in the logic model (see Fig 2). The implementation questions will demonstrate whether or not the interventions were delivered as intended and whether or not program outputs were achieved. The impact questions will demonstrate whether or not program outcomes and impacts were achieved and potentially provide information on the pathways between outcomes and impact.

#### Monitoring the implementation of DREAMS

As country teams developed their specific DREAMS program plans, they set targets for the number of recipients who would receive DREAMS interventions. Generally, country teams started by identifying the number of AGYW in each DREAMS district. Then, they explored factors that constitute vulnerability among this population, some of which were country specific (e.g., child marriage), and others that were more general (e.g., history of violence). They used available literature and administrative data to estimate the proportion of the AGYW population that was vulnerable based on these factors. Teams were expected to reach 50% of that vulnerable population over the first two years that they received DREAMS funding, which served as the basis for setting DREAMS targets.

PEPFAR program indicators are reviewed on a quarterly, semi-annual, or annual basis to monitor whether or not implementing partners are achieving their targets in DREAMS geographic areas (see Table 2). These indicators are age and sex disaggregated which serves as a measure of whether implementation is happening with the right sex and age groups. In addition to the quantitative program indicators, country teams submit qualitative narratives on their successes and challenges with identifying and engaging the most vulnerable AGYW, and layering the components of the DREAMS core package. Finally, as DREAMS partners, the Bill and Melinda Gates Foundation funded Population Council to conduct implementation science research on the barriers and facilitators of successful program implementation, including reaching the most vulnerable girls, reaching their male partners, and successfully implementing PrEP in this population.

**Table 2:** PEPFAR program indicators monitored for DREAMS.

Program Area	Indicator	Description	Disaggregation
Prevention*	GEND_GBV	Number of people receiving post gender-based violence clinical care based on the minimum package	violence type/ age/ sex
Know HIV Status*	HTS_TST	Number of individuals who receive HIV testing services and received their results	testing modality/ age/ sex/ result
Prevention*	KP_PREV	Number of key populations reached with individual and/or small group-level HIV prevention interventions	key population type/ sex
Prevention *	OVC_SERV	Number of beneficiaries served by PEPFAR orphans and vulnerable children programs for children and families affected by HIV	service modality/ age/ sex
Know HIV Status*	PMTCT_STAT	Percentage of pregnant women with known HIV status at antenatal care	status/ age
Prevention*	PREP_NEW	Number of individuals who have received oral antiretroviral pre-exposure prophylaxis to prevent HIV infection	key population type/ age/ sex
Prevention*	PP_PREV	Number of the priority populations reached with standardized HIV prevention interventions that are evidence-based	age/ sex
On ART**	TX_CURR	Number of adults and children currently receiving antiretroviral therapy	age/ sex
On ART**	TX_NEW	Number of adults and children newly enrolled on antiretroviral therapy	Key population type/ status/ age/ sex
Viral Suppression**	TX_RET	Percentage of adults and children known to be on treatment 12 months after initiation of antiretroviral therapy	Retention time in months/ age/ sex
Prevention**	VMMC_CIRC	Number of males circumcised as part of the voluntary male circumcision for HIV prevention program	Circumcision type/ age

* Interest for DREAMS is female-specific

**Interest for DREAMS is male-specific

#### Evaluating the impact of DREAMS

Changes in program outcomes and impacts as expressed in the DREAMS logic model are also being measured in multiple ways. In all 10 original DREAMS countries, modeling of new HIV diagnoses among 15–24 year old AGYW in DREAMS districts is being used to look at changes over time. This geo-statistical modeling utilizes program data from 90,000 antenatal clinics tracked on a quarterly bases, including new diagnoses among 15–24 year old females and pregnancy rates. The Bill and Melinda Gates Foundation also funded the London School of Hygiene and Tropical Medicine (LSHTM) to conduct impact evaluations in three DREAMS countries–Kenya, South Africa and Zimbabwe. LSHTM supplements existing surveillance systems to measure exposure to DREAMS programming, intermediate outcomes, as well as to track changes in incidence over time.

Due to the complex nature of the DREAMS core package of interventions and the fact that DREAMS is embedded within an environment that offers other programs and services to AGYW (e.g., other core PEPFAR activities, partner government activities), measuring the impact of DREAMS is also complex. No one data source can adequately describe the DREAM story over time, rather it will be critical to triangulate multiple sources of data. For example, program data on l will allow for a better understanding of data from the impact studies. This triangulation will give a much more nuanced picture of the successes of DREAMS, the accuracy of the theory of change, as well as those aspects of DREAMS programming that need to be improved.

### Challenges in DREAMS implementation

DREAMS has changed the way that PEPFAR and other donors approach programming for AGYW. HIV in AGYW was a public health emergency when DREAMS was created in 2014. Finding the most vulnerable girls has proven difficult for teams working in areas where girls are often transient, out of school, or under the control of a parent or partner who may not support their participation. Young women 20–24 are especially difficult to recruit and retain because of the multiple demands on their time, including work and child care. The practice of layering is new and challenging for DREAMS programs, given the level of coordination required. Currently, some countries are struggling to reach a majority of AGYW with 3 or more interventions. However, if AGYW are only receiving one to two services, they are not benefiting from all that DREAMS has to offer. Relatedly, measuring coverage and whether or not layering is occurring requires new indicators and monitoring systems. Although challenges exist, these lessons and feedback allow for continual improvements to DREAMS implementation.

### Conclusion

Substantial gains in reducing new HIV infections globally are at risk due to the disproportionate impact of the epidemic on AGYW. Several critical countries are progressing towards epidemic control based on newly released data from the PEPFAR-supported PHIAs [108]. Despite this progress, the PHIAs also show that we may lose ground due to the failure to stop new infections in AGYW. We must act now to curb these new infections by addressing the unique and often inequitable circumstances affecting the daily lives of AGYW. Biomedical approaches, alone, have not and cannot meaningfully reduce the spread of HIV for this priority population. However, incorporating biomedicine into comprehensive prevention programs that also address structural and behavioral factors, is likely to have a larger impact on reducing the number of new infections in the most vulnerable AGYW. Meeting the needs and demands of AGYW requires unpacking the data to identify challenges and risks for an individual girl or young woman. Once identified, then and only then, can a response be tailored to mitigate risks in a holistic way. The DREAMS Partnership provides the blueprint for doing exactly that by implementing youth-focused HIV prevention within the parameters laid out in the DREAMS core package. The “layered” approach ensures that multiple interventions and services at both the community and facility levels are provided to an AGYW. Some components of the core package are provided to all DREAMS recipients (e.g., school or community based HIV prevention education) whereas other components are based on an AGYW’s individual needs and circumstances (e.g., only survivors of violence are provided with post-violence care). The DREAMS core package not only educates, supports and empowers AGYW, but it also equips their families and communities with the necessary tools to support their positive development. DREAMS interventions and services work together to provide comprehensive HIV prevention to decrease risk and enhance supports at the individual, family and structural levels. The synergy of the components of the DREAMS core package are expected to change not only HIV acquisition, but also other outcomes in the lives of AGYW that are directly or indirectly related to their HIV risk (e.g., violence, early sex, early pregnancy, access to education). DREAMS is not a moment it is a movement—DREAMS is well positioned to change the narrative and course of the epidemic to achieve an AIDS-free future for AGYW and their communities.
